# Climatic factors determine the yield and quality of Honghe flue-cured tobacco

**DOI:** 10.1038/s41598-020-76919-0

**Published:** 2020-11-16

**Authors:** Zuoxin Tang, Lulu Chen, Zebin Chen, Yali Fu, Xiaolu Sun, Binbin Wang, Tiyuan Xia

**Affiliations:** 1 College of Agricultural and Life Sciences, Kunming University, Kunming, 650214 Yunnan China; 2 College of Forest Science, Beijing Forestry University, Beijing, 100083 China; 3 Honghe Branch of Yunnan Tobacco Company, Mile, 652300 Yunnan China; 4 Agronomy College, Qingdao Agricultural University, Qingdao, 266000 Shandong China; 5 Yunnan Iridium Biotechnology Co., Ltd., Kunming, 650031 Yunnan China

**Keywords:** Ecology, Climate sciences

## Abstract

Flue-cured tobacco (*Nicotiana tabacum* L.) is a major cash crop in Yunnan, China, and the yield, chemical components, and their proportions decide the quality of tobacco leaves. To understand the effects of environmental factors (soil and climatic factors) on the yield and quality of flue-cured tobacco and determine the main regulating factors, we selected three flue-cured tobacco cultivars [K326, Yunyan87 (Yun87), and Honghuadajinyuan (Hongda)] grown in the Honghe Tobacco Zone. Indices related to yield and economic traits, chemical component properties, soil physical and chemical properties, and climatic factors at different planting sites, were evaluated. We used variance analysis, correlation analysis, and redundancy analysis (RDA) in this study. The results showed that the yield and chemical component properties of flue-cured tobacco, except for the number of left leaves and plant total sugar (PTS) content, were significantly correlated with climatic factors. Particularly, the yield increased in drier and sunnier weather. In terms of the carbon supply capacity, PTS, petroleum ether (PPE), and starch contents (PS) were higher under high-altitude and high-latitude climatic conditions, whereas for the nitrogen supply capacity, plant nitrogen (PTN) and nicotine (PN) contents improved under low-altitude and low-latitude climatic conditions. PTS, reducing sugar (PRS), potassium (PTK), chlorine (PCL), and PPE contents were negatively related to soil clay content, soil pH, and soil organic matter, whereas PRS and PTK contents were positively correlated with alkali-hydrolyzed nitrogen (AN). According to RDA, the soil clay, AN, available phosphorus (AP), and soil chlorine content (SCL) strongly affected the quality of flue-cured tobacco. The quality of the K326 and Yun87 cultivars was mostly influenced by moisture, whereas the quality of the Hongda cultivar was mostly affected by temperature. In conclusion, compared with soil properties, climatic factors more significantly affect the yield and quality of Honghe flue-cured tobacco leaves.

## Introduction

Flue-cured tobacco (*Nicotiana tabacum* L.) is a major cash crop in Yunnan, China. Yunnan produces approximately 750,000 t of flue-cured tobacco on 320,000 ha of land every year^1^ and approximately 50% of the total tobacco leaf yield in China^2^. Because of its unique climate and geographical environment, Yunnan Province produces high-quality flue-cured tobacco that has been applauded for its golden color, fragrant aroma, mild effect, and pure taste^3^. Tobacco can adapt to a wide range of conditions but is very sensitive to the environment. The quality of tobacco is related to various factors, including the climate^4,5^, crop rotation patterns^6,7^, soil properties^8,9^, and soil microbes^10,11^. The natural environment is the ecological basis for the development of high-quality tobacco leaves.

Ecological conditions have great impacts on the yield and quality of tobacco^12^. In particular, tobacco needs certain light, temperature, water, and other climatic conditions to develop well^13^. In the process of planting and production, tobacco is susceptible to low-temperature stress, which causes early flowering of tobacco^14^ and can also induce gray speckles during flue-curing^15,16^, thus affecting the economic value of the crop^17^. High-temperature forced ripening also reduces the yield and quality of tobacco leaves^18^. Temperature plays a predominant role in affecting the rate of soil carbon mineralization^19^, and tobacco’s resistance mechanisms are also temperature dependent^20^. In addition, the properties of tobacco leaves, such as biomass, leaf area, sugar, nicotine content, etc., are largely influenced by moisture factors^21^. In Yunnan, 75% of the annual precipitation is concentrated between May and September^22^. Compared to other crop species, tobacco is relatively more sensitive to insufficient water supply. Studies have demonstrated that the total sugar, reducing sugar, total nitrogen, and total potassium contents are positively correlated with irrigation amount, whereas the contents of nicotine, protein, and chlorine are negatively related to irrigation amount in the growing area of flue-cured tobacco in Guizhou Province^23^. Studies also showed that the responses of the Yuyan6 and Yunyan87 (Yun87) varieties to water stress differed at the proteomic level^24^. Moreover, climatic factors such as rainfall, temperature, and frost can affect the incidence and severity of disease in flue-cured tobacco^4^. To improve the potential of tobacco cultivars, it is necessary to understand the characteristics of cultivars under different environmental conditions.

Soil physicochemical properties also play an important role in tobacco growth. Normal growth and maturation of tobacco are closely related to the physical and chemical properties of the tobacco planting soil, and simultaneously, tobacco requires appropriate amounts of various nutrients from the soil. Many studies have explored the effects of soil properties on tobacco, including studies on soil pH^7^, soil fertility^25^, and soil microbes^26^ in different tobacco-growing areas in China. For example, the organic matter and total nitrogen contents in soil should be suitable in regions of tobacco cultivation^27^. The lack of nutritional elements or imbalanced proportions will impede or cause abnormal changes in the growth and physiological metabolism of tobacco plants^8^. For example, high concentrations of total nitrogen induce excessive growth of tobacco^28^ and cause an imbalance of chemicals in the plants^29^, affecting the yield and quality of tobacco leaves. Moreover, the soil substrate is a determining factor for both aboveground and belowground biomass^19^. However, the combined effects of climatic factors and soil properties on the quality of tobacco leaves and their relative contributions remain unclear. In 2015, flue-cured tobacco in the Honghe Tobacco Zone was mainly planted in four counties (cities), Luxi (LX), Mile (ML), Jianshui (JS), and Shiping (SP) (Table 1), with crop areas located at high and low elevations with different climatic conditions. These four planting sites accounted for 80.97% of the total tobacco-growing area in Honghe. The main cultivars of flue-cured tobacco planted in these areas were the K326, Honghuadajinyuan (Hongda), and Yun87 cultivars, which comprised 39.33%, 35.67%, and 25.00% of total production, respectively. In this study, using the collected data, we aimed to address the following two questions. (1) Do the yield and quality of these three flue-cured tobacco cultivars differ among the four planting sites? And, (2) what are the dominant environmental variables (climatic or soil factors) explaining the yield and quality of different flue-cured tobacco cultivars?

**Table 1:** Basic information of flue-cured tobacco planting sites.

Sites	Jianshui (JS)	Luxi (LX)	Mile (ML)	Shiping (SP)
Elevation	1217–2051	1491–2071	1206–2154	1380–2198
Longitude	23.39–24.05	24.19–24.72	23.90–24.56	23.40–24.03
Latitude	102.38–103.13	103.39–104.01	103.16–103.63	102.22–102.67
Soil type	Red soil, yellow soil, paddy soil	Red soil, yellow soil, paddy soil	Red soil, yellow soil, clay soil, purple soil	Red soil, yellow soil, paddy soil
Tobacco area (ha)	5866.67	11,866.67	11,866.67	6833.33
Tobacco area ratio (%)	13.04	26.37	26.37	15.19
Field rainfall (mm)	573.38 ± 4.47	662.01 ± 3.73	579.13 ± 1.30	589.45 ± 5.18
Field temperature (℃)	18.1 ± 0.16	14.8 ± 0.10	16.5 ± 0.19	17.1 ± 0.23
Field sunshine hours (h)	623.40 ± 1.01	605.14 ± 0.82	625.11 ± 0.37	626.04 ± 0.76
Field relative abundance (%)	76.60 ± 0.05	78.81 ± 0.04	77.25 ± 0.05	76.97 ± 0.03
Clay (%)	54.46 ± 2.25	61.42 ± 1.26	59.18 ± 1.20	55.24 ± 2.38
pH	6.19 ± 0.16	6.69 ± 0.10	6.29 ± 0.11	5.52 ± 0.15
SOM (g/kg)	19.80 ± 1.81	25.65 ± 1.25	24.40 ± 1.16	23.78 ± 1.40
AN (mg/kg)	90.35 ± 7.82	102.47 ± 4.60	105.85 ± 4.49	109.17 ± 6.53
AP (mg/kg)	32.59 ± 5.01	30.78 ± 3.02	31.64 ± 3.45	37.45 ± 5.69
AK (mg/kg)	203.57 ± 16.84	218.60 ± 11.34	201.60 ± 10.16	188.72 ± 14.26
CL^-1^ (mg/kg)	11.78 ± 2.39	5.69 ± 0.67	7.69 ± 0.77	12.00 ± 1.74

The elevation, the longitude and the latitude is the range value of the sampling samples in Jianshui (JS), Luxi (LX), Mile (ML) and Shiping (SP) these four study sites. Soil type is determined by the classification of Chinese Soil Taxonomy (CST); Tobacco area is the planting area of flue-cured tobacco in Jianshui (JS), Luxi (LX), Mile (ML) and Shiping (SP) these four study sites; Tobacco area ratio is the proportion of the above planting areas that accounted for the total planting area in Hani-Yi Autonomous Prefecture of Honghe in 2015; the field rainfall, field temperature, field sunshine hours and field relative humidity mean the variation of rainfall, temperature, sunshine hours and relative humidity in the field time (from May to August). Soil clay, soil pH, soil organic matter (SOM), soil alkali-hydrolyzed nitrogen (AN), soil available phosphorus (AP), available potassium (AK) and soil chlorine content (SCL), these data in the table are the means ± standard error.

## Materials and methods

### Experimental location

**Figure 1:**
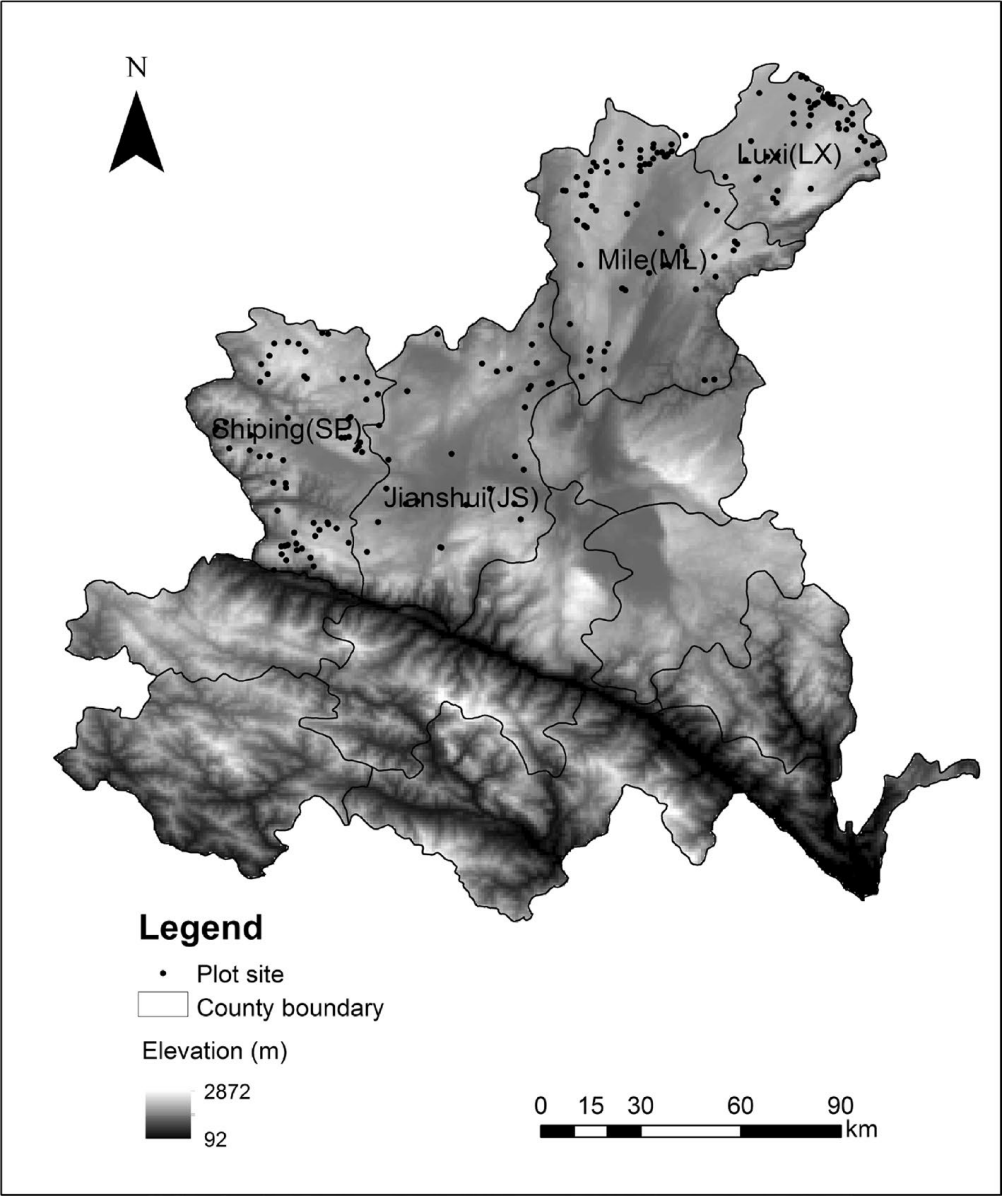
Distribution of soil samples of four main tobacco-growing sites [Jianshui (JS), Luxi (LX), Mile (ML) and Shiping (SP)] in Hani-Yi Autonomous Prefecture of Honghe. The elevation data were obtained from the Global Map data archives (https://globalmaps.github.io/), the spatial coordinate system is GCS-WGS84, with a 1-km resolution, and plot site distribution was according to our sampling point, with ArcGIS10.1.

The experiment was conducted in the Hani-Yi Autonomous Prefecture of Honghe (22.43–24.75° N, 101.78–104.27° E, referred to as Honghe in the following section), Yunnan Province, China, an area belonging to the plateau subtropical monsoon climate region. The average annual rainfall is 1491 mm. The average annual sunshine is 1065–2300 h. In the middle of March, the temperature stabilizes, remaining above 13.0 ℃, then rises to 19.0–24.5 ℃ in April. The average temperature in May–August is above 20.0 ℃, and the average temperature in September is between 20.4 and 24.6 ℃, which is the most suitable temperature for flue-cured tobacco growth. The three main local cultivars (K326, Yun87, and Hongda) were used in the experiment and were planted in JS, LX, ML, and SP (Fig. 1). The soils were defined as red soil, yellow soil, paddy soil, clay soil, and purple soil (Chinese Soil Taxonomy), and the average concentrations of clay, pH soil organic matter (SOM), alkali-hydrolyzed nitrogen (AN), available phosphorus (AP), available potassium (AK), and soil chlorine (SCL) are shown in Table 1.

### Field management

Cultural practices for Virginia tobacco production include choosing appropriate varieties, planning effective pest-control measures, and deciding the best time to top or harvest a crop; in particular, breeding seedlings, transplanting, fertilizing, field management, removing the apical meristems (flowers), crop rotation, and management of plant diseases and pest insects. These practices were recommended by the Tobacco Institute of Yunnan and are used in other Yunnan areas^30^. The specific cultural practices applied in this study were as follows.

#### Breeding seedlings

Floating breeding to obtain healthy and disease-free tobacco seedlings started from March 1 to 10.

#### Transplanting

Transplanting under plastic film occurred from April 10 to 20. The transplanted seedlings were 5–7 cm in height with 4–5 leaves combined on one core. The transplant spacing for all cultivars was 120 cm × 50 cm, with a final density of 15,000–16,500 plants per ha.

#### Irrigation

In 2015, irrigation methods included water cellars (41.67%), dammed ponds (19.00%), reservoirs (11.00%), rivers (11.00%), and mountain springs, water wells, and vauclusian springs (7.34%); 9.99% of the fields had no water conservancy facilities.

#### Crop rotation

The following are the main flue-cured tobacco rotation modes in the region: flue-cured tobacco → corn → flue-cured tobacco, flue-cured tobacco → wheat → flue-cured tobacco, flue-cured tobacco → rice → flue-cured tobacco, and flue-cured tobacco → others → flue-cured tobacco. In 2015, the flue-cured tobacco → corn → flue-cured tobacco mode was most frequently employed (75.33% of production).

#### Fertilizing

Pure nitrogen fertilizer was applied at a rate of 90–110 kg/ha for both the K326 and Yun87 cultivars, but with N:P_2_O_5_:K_2_O ratios of 1:1:2.5–3.0 and 1:1:3.0–3.5, respectively. For the Hongda cultivar, pure nitrogen fertilizer was applied at a rate of 60–75 kg/ha with a N:P_2_O_5_:K_2_O ratio of 1:1:3.0–5.0.

#### Plant diseases and pest insect management

Black shank disease, root knot nematode disease, viral diseases, cutworms, *Prodenia litura* (Fabr.), and *Myzus persicae* (Sulzer) have caused heavy losses in Honghe. Disease-control measures mainly include physical measures, chemical measures, and biological measures, such as crop rotation, disease-resistant cultivar selection, chemical pesticides, application of organic fertilizer, and deep turning (30 cm) and drying of field soil, while following the principle that biological control is the focus, physical control is auxiliary, and chemical control is supplementary.

#### Picking and baking

To enable optimal growth and development of flue-cured tobacco, the lowest leaves (lugs) and the apical meristem must be removed, and the remaining effective leaves are known as “left leaves.” The numbers of left leaves per plant were 18–20, 16–18, and 18–20 for the K326, Hongda, and Yun87 cultivars, respectively. The squaring stage was around June 5 for K326 and Yun87, and around May 30 for Hongda; the height after removing the apical meristem was 100–110 cm for the K326 and Yun87 cultivars and 90–110 cm for the Hongda cultivar. The leaf samples were weighed to calculate yields, which were 10.67–12.01, 8.67–10.01, and 10.01–11.34 kg/ha for K326, Hongda, and Yun87, respectively. As flue-cured tobacco grows and matures, the fresh leaves turn yellow and mature layer-by-layer from the bottom of the plant. Two to three effective mature lower and middle leaves are harvested at a time, whereas four to six upper leaves are harvested at a time. The harvesting stage for the K326, Yun87, and Hongda cultivars was from June 30 to September 10. In our study, we collected the middle leaves in August after cultivating the plants for 85–95 days and topping for 30–40 days. At this time, the tobacco leaves were pale yellow, and 80% of the leaves had yellowed and were exhibiting white, bright main veins, white branch veins, downward rolled leaf apices and leaf margins, and marked mature plaques; mature leaves were collected on two to three occasions. After the tobacco leaves are picked, the leaf samples should be baked for a few days under different temperatures using an intelligent baking method.

#### Grade classification

According to the National Standard (GB2635-1992), the grades of flue-cured tobacco were classified based on seven appearance grade factors, including maturity, leaf structure, identity, oil content, color, length, and residual injury, which were divided into 42 levels in total. For the sake of statistics, X, C, and B represent the lower, middle, and upper tobacco leaves, respectively; L and F represent the colors lemon and orange. Middle tobacco leaves of grade 3 with an orange color (C3F) were used for the analysis of chemical components in our study. The proportion of superior middle tobacco (both middle and superior grades, Prop) and the proportion of superior tobacco (only superior grades) were estimated based on national purchasing data and data on the supervision and inspection of grade quality during the industry–commerce handover in that year. Additionally, each grade had an associated monetary value; this value was multiplied with crop yield to estimate the overall crop economic value (Economic value).

### Soil and plant sampling

We collected 39, 76, 87, and 42 soil samples from JS, LX, ML, and SP, respectively (Fig. 1). The sampling points were determined according to tobacco cultivar, soil type, tobacco area, and altitude factors. A GPS was used to mark the location of and label each sampling point, where the 0–20 cm soil layer was collected according to the "S" type sampling method before seedlings were transplanted; ten subsamples were collected between and pooled to make up a 1-kg soil sample^31,32^. Gravel, roots, and large organic residues were manually removed before the soil samples were passed through a 2 mm sieve, then samples were stored in sealed bags in an ice cooler within 2 h of collection. In the laboratory, the samples were used to measure soil mechanical composition and soil properties (e.g., carbon, nitrogen, and pH). Tobacco plant samples of the three cultivars (K326, Yun87, and Hongda) were also collected from the soil sampling sites.

### Meteorological data collection

The Honghe branch of the Yunnan Tobacco Company has access to 30 years (1980–2010) of meteorological data collected by the Yunnan meteorological observatory, including monthly temperature, rainfall, sunshine, and average relative humidity data. We also obtained monitoring data on temperature and precipitation as well as other meteorological data for 2013–2015. The data were standardized using an algorithm to obtain temperature, sunshine, rainfall, and humidity data for our analysis^33^. We calculated field temperatures, sunshine hours, rainfall, relative humidity from May to August. Sunshine hours and rainfall were calculated as sum values, and temperature and relative humidity were calculated as average values; the values for all parameters are summarized in Table 1.

### Measurement of plant nutrient elements

C3F leaf samples were used to determine the final chemical compositions. The plant total nitrogen (PTN), potassium (PTK), and chlorine (PCL) were analyzed according to the YC/T161-2002, YC/T 173-2003, and YC/T 162-2011 methods, respectively. Plant total sugar (PTS) and reducing sugar (PRS) contents were analyzed according to the YC/T 159-2002 method. Plant nicotine (PN), starch (PS), and petroleum ether (PPE) contents were analyzed according to the LY/T1228-2015, NY/T1121.7-2014, and NY/T889-2004 methods, respectively. In this study, we also calculated the plant sugar/alkaloid (PSA) ratio and the total nitrogen to total alkaloids (PNA) ratio.

### Soil physicochemical properties

Soil mechanical composition (clay, < 0.001 mm) was calculated according to the NY/T1121.3-2006 method, and soil pH according to the NY/T1377-2007 method. SOM, AN, AP, AK, and SCL were analyzed according to the NY/T1121.6-2006, LY/T1228-2015, NY/T1121.7-2014, NY/T889-2004, and NY/T1378-2007 methods, respectively.

### Data analysis

The normality and homogeneity of the residuals of the predicted values were checked in terms kurtosis and skewness, respectively. It was necessary to use rank cases to transform the yield, proportion, and SCL variables to meet parametric assumptions of normality and homogeneity. Significant differences among treatments were tested using analysis of variance (ANOVA) followed by Duncan’s test of significant differences at a significance level of *p* < 0.05. Pearson correlation coefficients were used to describe the relationships between chemical composition of the tobacco leaves and soil and climatic factors. Furthermore, we carried out redundancy analysis (RDA) to determine the linkages between tobacco yield and quality and ecological factors^19^. The forward selection procedure in RDA, based on Monte Carlo permutation with 499 iterations, was performed to determine the most significant discriminating variables for tobacco yield and quality variables, and the significant variables (*p* < 0.05) are shown in the figures. Significance tests for RDA were performed using Canoco for Windows 4.5 (Biometris Plant Research International, Wageningen, The Netherlands). All statistical analyses were performed using SPSS version 17.0 (SPSS Inc., Chicago, IL, USA).

## Results

### Yield and quality of flue-cured tobacco

Based on the yield and economic value per hectare, as well as the proportion of superior middle tobacco and abundance of left leaves of flue-cured tobacco, as shown in Table 2, the tobacco cultivar interacted with planting site to influence yield and quality. In general, the abundance of left leaves and proportion of superior middle tobacco produced by the K326 cultivar were 0.30–3.01% and 3.99–11.29% higher than those produced by the other two cultivars, respectively, but the yield and economic value were 1.32–2.85% and 3.9–22.72% lower, respectively. However, the opposite was true for the Hongda cultivar; the abundance of left leaves and proportion of superior middle tobacco were lower by 2.62–2.92% and 6.56–10.14%, respectively, but the economic value was higher by 19.16–22.72%. The proportion of superior middle tobacco did not differ among the four planting sites when the K326 or Yun87 cultivar was planted. However, the proportion of superior middle tobacco was higher at the JS and SP planting sites compared to the LX and ML sites when the Hongda cultivar was planted. In addition, the yield and economic value were higher when K326 or Yun87 was planted at the ML site, with the yield increasing by 8.00–18.18% and the economic value increasing by 7.54–16.23%; however, K326 had more left leaves compared to Yun87.

**Table 2:** Variations of tobacco-plant production properties.

	Yield (kg/ha)	Economic value (USD/ha)	Prop (%)	Left leaves
**K326**				
JS	9.23 ± 0.21b	36.03 ± 0.17b	95.83 ± 0.67a	21.25 ± 0.23c
LX	8.13 ± 0.06a	36.41 ± 0.40b	94.54 ± 0.28a	19.19 ± 0.18a
ML	9.05 ± 0.18b	39.27 ± 0.67c	93.49 ± 1.19a	19.87 ± 0.21b
SP	8.38 ± 0.15a	34.19 ± 0.91a	94.97 ± 1.10a	18.80 ± 0.25a
**Hongda**				
JS	9.04 ± 0.18b	46.43 ± 0.94bc	93.97 ± 0.34b	18.27 ± 0.18b
LX	8.83 ± 0.11b	47.74 ± 0.84c	91.25 ± 0.85a	18.24 ± 0.17b
ML	8.82 ± 0.03b	44.09 ± 0.26ab	91.18 ± 0.27a	16.64 ± 0.15a
SP	8.30 ± 0.18a	42.09 ± 1.33a	95.74 ± 0.91b	18.31 ± 0.12b
**Yun87**				
JS	8.34 ± 0.01a	36.69 ± 0.72a	92.88 ± 0.48a	20.13 ± 0.13b
LX	8.14 ± 0.01a	36.85 ± 0.79a	92.91 ± 0.27a	19.50 ± 0.37a
ML	9.62 ± 0.16b	39.63 ± 0.62b	94.68 ± 0.79a	18.21 ± 0.27a
SP	8.39 ± 0.02a	36.32 ± 0.87a	95.53 ± 0.88a	19.20 ± 0.39ab
**Statistical analysis**				
Site	25.43 (< 0.001)	10.05 (< 0.001)	5.97 (< 0.001)	23.20 (< 0.001)
Cultivar	0.632 (0.534)	158.41 (< 0.001)	6.94 (0.001)	79.44 (< 0.001)
Site × cultivar	9.25 (< 0.001)	6.94 (< 0.001)	3.32 (0.004)	11.80 (< 0.001)

The yield is the weight of leaves samples; the Economic value is a value that flue-cured tobacco’s value multiplies with crop yield to establish overall crop economic value; Prop is the proportion of superior middle tobacco (middle grade levels plus superior grade levels); left leaves is the number of remaining effective leaves after removing the lowest leaves (lugs) and the apical meristem of flue-cured tobacco. Data in the table are the means ± standard error, and different letters indicate significant differences at 5% under Duncan’s test.

The variations in tobacco plant quality properties as influenced by cultivar type and planting site were analyzed by two-way ANOVA (Table 3). The PN, PRS, PPE, PS, and PNA in tobacco leaves were significantly affected by the interaction between cultivar type and planting site. Although there were no inter-site differences in PN in Yun87 when it was planted at the four sites, K326 and Hongda had higher PN contents at the JS and SP sites compared to the LX and ML sites. Although the PRS in K326 did not differ among the four planting sites, Hongda and Yun87 had higher PRS contents at ML and SP compared to JS and LX. PPE contents in all three cultivars were lowest at the LX site. The PSA ratio was > 11 and the PNA ratio was < 1 for all three cultivars, and K326 had the highest average PSA and PNA ratios. Both the PTK and PCL contents in tobacco were significantly influenced by cultivar and planting site but were not affected by the interaction between the two factors. PTK was higher and PCL was lower at the ML and SP sites. For all cultivars, the trend in PTK content followed that in PCL content, with the contents ranked in the following order: K326 > Hongda > Yun87. The PTN and PSA contents in tobacco were only significantly influenced by planting site. PTN was higher and PSA was lower at the JS and SP sites, whereas PTN was lower and PSA was higher at the LX and ML sites.

**Table 3:** Variations of tobacco-plant quality properties.

	PTN (%)	PN (%)	PTS (%)	PRS (%)	PTK (%)	PCL (%)	PPE (%)	PS (%)	PSA	PNA
**K326**										
JS	2.15 ± 0.08b	3.04 ± 0.09c	33.41 ± 0.85b	26.31 ± 0.69a	1.71 ± 0.08b	0.44 ± 0.10ab	5.42 ± 0.21ab	3.38 ± 0.29a	11.17 ± 0.50a	0.71 ± 0.02a
LX	1.82 ± 0.05a	2.36 ± 0.11ab	35.28 ± 0.54b	27.96 ± 0.55a	1.41 ± 0.06a	0.62 ± 0.09b	4.97 ± 0.14a	4.92 ± 0.34b	16.33 ± 1.01b	0.80 ± 0.02ab
ML	1.81 ± 0.06a	2.18 ± 0.11a	35.42 ± 0.75b	28.09 ± 0.47a	1.89 ± 0.05b	0.32 ± 0.04a	5.21 ± 0.11ab	4.08 ± 0.29ab	18.60 ± 1.72b	0.87 ± 0.04b
SP	2.09 ± 0.10b	2.67 ± 0.10b	31.04 ± 1.03a	27.38 ± 0.82a	1.94 ± 0.10c	0.29 ± 0.08a	5.54 ± 0.15b	3.89 ± 0.60ab	11.99 ± 0.76a	0.78 ± 0.03ab
**Hongda**										
JS	2.04 ± 0.07a	2.58 ± 0.16ab	33.83 ± 0.67a	27.40 ± 0.61ab	1.61 ± 0.07a	0.33 ± 0.05ab	5.35 ± 0.20ab	5.38 ± 0.57b	13.86 ± 0.88ba	0.82 ± 0.04a
LX	1.86 ± 0.08a	2.36 ± 0.12a	33.85 ± 0.83a	27.05 ± 0.61a	1.44 ± 0.05a	0.33 ± 0.04b	5.00 ± 0.08a	4.67 ± 0.33ab	15.76 ± 0.96a	0.81 ± 0.02a
ML	1.86 ± 0.06a	2.45 ± 0.11a	34.48 ± 0.83a	29.41 ± 0.60b	1.64 ± 0.09a	0.24 ± 0.04ab	5.39 ± 0.12ab	4.77 ± 0.34ab	15.48 ± 1.17a	0.79 ± 0.02a
SP	2.06 ± 0.09a	2.90 ± 0.16b	33.12 ± 1.14a	29.11 ± 0.66ab	1.93 ± 0.09b	0.18 ± 0.02a	5.65 ± 0.18b	3.61 ± 0.43a	12.36 ± 1.21a	0.73 ± 0.04a
**Yun87**										
JS	2.17 ± 0.08b	3.01 ± 0.19a	32.66 ± 1.13a	25.75 ± 1.23ab	1.58 ± 0.16a	0.18 ± 0.02a	6.60 ± 0.28b	3.41 ± 0.21a	11.38 ± 1.25a	0.74 ± 0.04a
LX	1.97 ± 0.14b	2.82 ± 0.23a	31.99 ± 0.97a	24.87 ± 0.90a	1.37 ± 0.09a	0.29 ± 0.05a	5.46 ± 0.22a	4.11 ± 0.34ab	12.13 ± 1.08a	0.71 ± 0.03a
ML	1.71 ± 0.06a	2.21 ± 0.12a	34.53 ± 0.81a	27.81 ± 0.61bc	1.93 ± 0.09a	0.25 ± 0.02a	5.70 ± 0.14a	4.48 ± 0.33ab	16.77 ± 1.01b	0.81 ± 0.04a
SP	2.11 ± 0.11ab	2.93 ± 0.21a	34.75 ± 1.06a	29.79 ± 0.88c	1.78 ± 0.15a	0.24 ± 0.05a	5.47 ± 0.19a	4.82 ± 0.55b	12.61 ± 1.22a	0.73 ± 0.02a
**Statistical analysis**										
Site	10.67 (< 0.001)	11.63 (< 0.001)	2.59 (0.54)	7.34 (< 0.001)	13.34 (< 0.001)	2.95 (0.034)	7.04 (< 0.001)	0.92 (0.431)	10.53 (< 0.001)	3.07 (0.0281)
Cultivar	0.14 (0.872)	1.41 (0.247)	0.12 (0.891)	2.59 (0.78)	3.04 (0.050)	3.70 (0.026)	9.16 (< 0.001)	2.00 (0.137)	0.88 (0.417)	2.02 (0.134)
Site × cultivar	0.87 (0.518)	2.25 (0.039)	2.05 (0.059)	2.28 (0.037)	1.03 (0.409)	1.28 (0.268)	2.39 (0.019)	2.59 (0.019)	2.08 (0.057)	2.33 (0.033)

PTN, PN, PTS, PRS, PTK, PCL, PPE, PS, is the content of nitrogen, nicotine, total sugar, reducing sugar, potassium, chlorine, petroleum ether and starch content of leaves. We also showed the ratio of sugar/alkaloid (PSA) and the ratio of total N to total alkaloids (PNA) in this table. Values are means ± standard error. Means followed by the same letter for a given factor are not significantly different at *P* < 0.05 (Duncan’s test).

### Correlations between yield and quality of flue-cured tobacco and environmental factors

Pearson correlation analysis was used to examine the relationships between the yield and internal chemical composition of flue-cured tobacco and soil properties and ecological factors (Table 4). The results showed that soil properties and climatic factors altogether influenced seven yield variables and eleven variables related to internal chemical composition in flue-cured tobacco. In particular, the yield of tobacco was negatively correlated with field rainfall and field relative humidity, but positively correlated with field temperature and field sunshine hours. The proportion of superior middle tobacco and number of left leaves were influenced by field temperature and SOM content. In addition to PTS, climatic conditions had significant effects on the chemical composition of tobacco leaves. PTK, PCL, and PPE contents were significantly correlated with the four climatic conditions. PSA and PNA contents were negatively correlated with field temperature and positively correlated with field relative humidity. Rainfall was negatively correlated with PTN and PRS contents but positively correlated with PS content. Field sunshine hours were positively correlated with PN and PRS contents. PTS, PRS, PTK, and PPE contents were negatively correlated with soil pH, and PRS and PTK contents were positively correlated with soil AN content. PCL and PPE contents were negatively correlated with soil physical clay and positively correlated with SCL content.

**Table 4:** Relativity between yield and chemical compositions of tobacco leaves and major ecological factors.

	Clay (%)	pH	SOM (g/kg)	AN (mg/kg)	AP (mg/kg)	AK (mg/kg)	SCL (mg/kg)	FR (mm)	FT (℃)	FS (h)	FH (%)
Yield (kg/ha)								− 0.548**	0.185**	0.535**	− 0.394**
Proportion (%)					− 0.169**				0.128*		
Left leaves			− 0.139*								
PTN (%)								− 0.130*			− 0.149*
PN (%)									0.203**	0.128*	− 0.201**
PTS (%)		− 0.133*									
PRS (%)		− 0.126*		0.145*				− 0.135*		0.201**	
PTK (%)		− 0.411**		0.150*			0.152*	− 0.333**	0.274**	0.346**	− 0.321**
PCL (%)	− 0.127*						0.216**	0.133*	− 0.195**	− 0.213**	0.202**
PPE (%)	− 0.243**	− 0.174**	− 0.200**				0.167**	− 0.288**	0.385**	0.294**	− 0.329**
PS (%)								0.134*	− 0.210**		
PSA									− 0.150*		0.171**
PNA									− 0.237**		0.185**

Soil clay content (clay), soil pH (pH), soil organic matter (SOM), soil alkali-hydrolyzed nitrogen (AN), soil available phosphorus (AP), available potassium (AK), soil chlorine content (SCL), and the rainfall (FR), temperature (FT), sunshine hours (FS) and relative humidity (FH) in the field time (from May to August). The yield is the weight of leaves samples; Prop is the proportion of superior middle tobacco (both middle and superior grades); Left leaves is the number of remaining effective leaves after removing the lowest leaves (lugs) and the apical meristem of flue-cured tobacco. *PTN* the total nitrogen content, *PN* the nicotine content, *PTS* the total sugar content, *PRS* the reducing sugar content, *PTK* the content of potassium, *PCL* the chlorine contents, *PPE* the petroleum mether content, *PS* the content of starch, *PSA* the ratio of total sugar to total alkaloids and PNA, the ratio of total N to total alkaloids. Asterisks denote statistically significant Pearson correlation analysis.

*^,^**P value ≤ 0.05, 0.01 level (bilateral), respectively.

### Factors that influence the yield and quality of flue-cured tobacco

**Figure 2:**
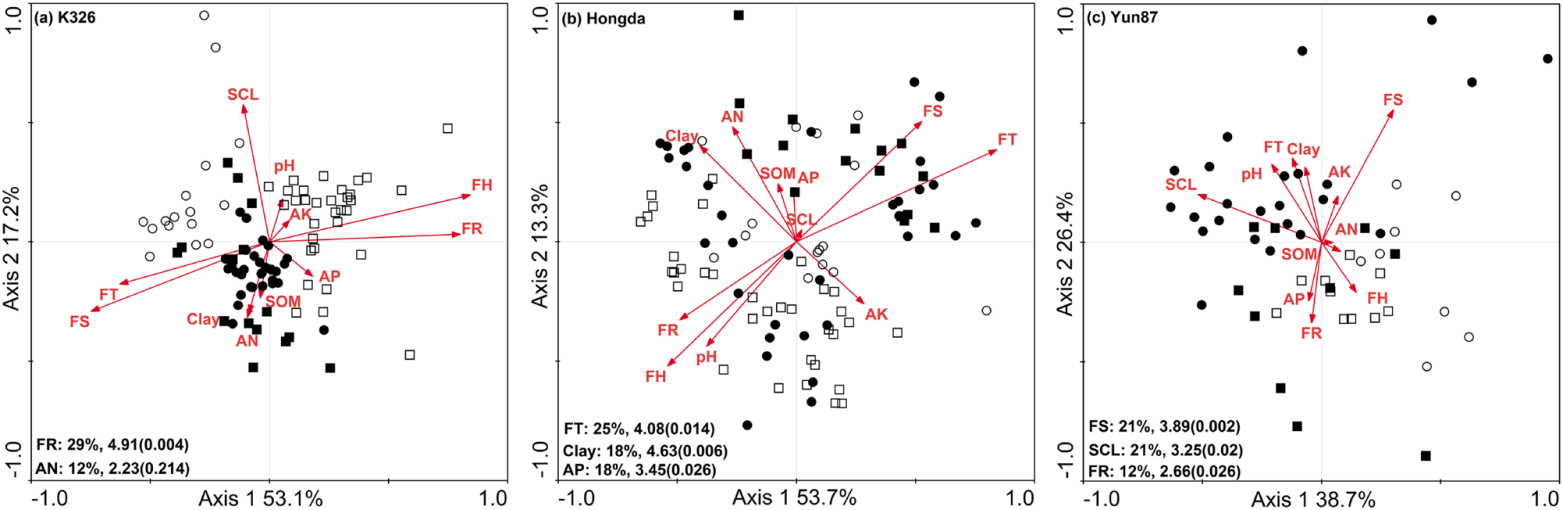
Redundancy analysis (RDA) of quantity and quality of different flue-cured tobacco cultivars and environmental factors. (**a**) K326 cultivar; (**b**): Hongda cultivar; (**c**): Yun87 cultivar. Empty circle, Jianshui site; square, Luxi site; solid circle, Mile site; solid square, Shiping site. RDA (Redundancy Analysis), a kind of PCA analysis constrained by environmental factors, could reflect samples and environmental factors in the same two-dimensional ranking diagram, from which the relationship between sample distribution and environmental factors could be intuitively showed. The environmental variables: *clay* soil physical clay, *pH* soil pH, *SOM* soil organic matter, *AN* the content of soil alkali-hydrolyzed nitrogen, *AP* available phosphorus content, *AK* available potassium content, *SCL* soil chlorine content, *FR* field rainfall, *FT* field temperature, *FS* field sunshine, *FH* field relative humidity.

Further RDA analysis showed that axes 1 and 2 explained 53.1% and 17.2% of the variation in the yield and quality of the K326 cultivar, respectively. Forward selection of the environmental factors in the RDA ordination showed that field rainfall and AN content explained 29% and 12% of the variation, respectively (Fig. 2a). For the Hongda cultivar (Fig. 2b), axes 1 and 2 explained 53.7% and 13.3% of the variation in yield and quality, respectively, and field temperature, clay content, and AP content were the most significant discriminating variables, accounting for 25%, 18%, and 18% of the variation, respectively. Cumulatively, the first two RDA axes explained 69.1% of the variance in the yield and quality of the Yun87 cultivar. Among the seven soil properties and four ecological factors, field rainfall, field sunshine hours, and SCL content were significantly correlated with tobacco yield and quality, explaining 21%, 12%, and 21% of the variation, respectively (Fig. 2c). All three plant cultivars were significantly affected by field rainfall and field relative humidity at the LX site. Meanwhile, the K326 and Hongda cultivars were also affected by soil pH and AK content, whereas the Yun87 cultivar was affected by AP and SOM contents at the LX site. At the ML site, all three cultivars were affected by field sunshine hours and field temperature. At the SP site, the sampling points were relatively dispersed, which might have affected the analysis of AN, SOM and SCL contents. At the JS site, K326 yield and quality were negatively correlated with AP content, whereas those of Hongda and Yun87 were correlated with field relative humidity (Fig. 2).

## Discussion

### Changes in the yield and economic value of tobacco leaves

Many factors affect the quality of tobacco leaves, and as a cash crop, the yield is an important index^32,34^. Crop yield is affected by various factors, such as site location, soil properties, solar radiation, precipitation, and temperature^35,36^. Sunshine hours in the range of 500–700 h are suitable for tobacco^37^, hence our study area was suitable with field sunshine hours ranging from 568 to 638 h. In this study, the field sunshine hours and field temperature were highest at the JS site and lowest at the LX site, and trends in yield were consistent with this tendency. The yields of flue-cured tobacco improve in sunnier and drier weather^38^. Overall, from our results, the yield was negatively correlated with field rainfall and field relative humidity, and positively influenced by field sunshine and field temperature. In this study, the proportions of superior middle tobacco and superior tobacco were 91.18–95.83% and 62.19–73.29%, respectively, which is above the theoretical maximum proportion of superior tobacco (60%)^39^. A previous study demonstrated that the removal of four leaves per plant results in a moderate yield of flue-cured tobacco during the tobacco growing season^9^. In this study, the number of left leaves of K326 and the proportion of superior tobacco were higher, whereas the yield and economic value were lower, while the results of the Hongda cultivar were the opposite; this may indicate that the Hongda cultivar is more suited to the Honghe tobacco-planting area. However, both the yield and economic value of flue-cured tobacco were affected by the interaction between tobacco cultivar type and planting site; K326 was more suited to the JS site, and there were no significant differences between K326 and Yun 87 at the ML site, with both producing higher yields with a higher economic value.

### Quality differences in flue-cured tobacco leaves

The chemical components in tobacco leaves and their proportions also determine the quality of tobacco leaves^40^, thus exerting significant influence on the smoking quality of cigarettes^41^.

#### Effects of environmental factors on nitrogen and nicotine contents in tobacco leaves

Nitrogen deficiency is cited as “the most common deficiency in tobacco”^42^ and reduces the yield and quality of tobacco leaves^43^. Available nitrogen is needed to sustain full growth until flowering, which can directly influence the yield of flue-cured tobacco^44^, but excessive nitrogen will reduce the content of phenolic substances and lignin in plants and weaken the disease resistance of tobacco plants^45^. Even after the flowering stage, nitrogen overfertilization leads to a reduction in quality and economic value and gives rise to nitrogen pollution in the soil and underground water^44^. Overall, nitrogen contents in tobacco leaves were higher at the JS and SP sites, lower at the LX and ML sites, and negatively correlated with field rainfall and field relative humidity. Total nitrogen and alkaloid contents are used to reflect nitrogen supply capacity^43^. The presence of alkaloids significantly affects the taste of tobacco, and a nicotine content that is too high can increase the biting taste of tobacco^46^. The nicotine content was highest in the Yun87 cultivar, and nicotine contents were higher at the JS and SP sites than at the LX and ML sites. The nicotine content was positively influenced by field temperature and field sunshine hours but negatively affected by field relative humidity in this study. The nicotine content in tobacco can be increased by 1 g/kg and 3.3–3.5 g/kg by a 1.0 °C increase in the ground temperature at 0–5 cm and the air temperature, respectively^47^; thus, to some extent, temperature increases can stimulate an increase in nicotine content in tobacco.

#### Effects of environmental factors on the potassium content in tobacco leaves

The potassium content in tobacco leaves is considered an important indicator of high quality. A high potassium content in tobacco leaves can improve the combustibility, aroma, taste, and safety, and the potassium content plays a key role in the appearance and internal quality of tobacco leaves^48^. Potassium deficiency leads to a significant decline in plant growth and yield, and a low potassium content in flue-cured tobacco is always a matter of concern in tobacco production in China. Overall, the potassium content in the Yun87 cultivar did not differ significantly among the four sites, whereas potassium contents in K326 and Hongda were the highest at the SP site. The results of this study are consistent with reports that potassium content is negatively correlated with soil pH and positively correlated with AN^49^. However, climatic factors, not soil factors, were the significant factors influencing potassium content in this study; the potassium content was positively correlated with field sunshine hours and field temperature but negatively correlated with field rainfall and field relative humidity. Slightly acidic soil conditions, improved soil physical and chemical properties, and appropriate soil nitrogen content can promote the absorption and accumulation of potassium in tobacco, improving the potassium content. Although potassium deprivation in soil has been shown to lead to large reductions in plant growth and yields^50^, in our study, the yield was not influenced by AK content.

#### Effects of environmental factors on the chlorine content in tobacco leaves

Flue-cured tobacco is a chlorine-free crop, and a chlorine content that is too high or too low is bad for the growth of tobacco plants. When the chlorine content is too low, the tobacco leaves are easily broken, affecting the quality. Relatively large amounts of chloride have many adverse effects on the quality of tobacco leaves, resulting in effects such as a poor burning capacity, muddy appearance, and undesirable odor as well as high hygroscopicity, which causes discoloration during storage^50^. In our study, the K326 cultivar had the highest chlorine content, and chlorine contents were highest at the LX site, which was significantly influenced by the SCL content. Chlorine content was also positively correlated with field rainfall and field relative humidity but negatively correlated with field sunshine hours and field temperature. Chlorine in tobacco leaves is mainly absorbed from soil, while the chlorine input to the soil is primarily from water and fertilizer, mostly from previous crops and irrigation water in China^51^.

#### Effects of environmental factors on the sugar content in tobacco leaves

Sugar content is an important index used to evaluate the quality of tobacco and can also reflect the carbon supply capacity^52^. A sugar content that is too low or too high can influence the taste, likely resulting in a more biting or acidic taste^53,54^. Based on the analysis of our results, PTS contents in the Hongda and Yun87 cultivars did not differ among the four planting sites, whereas the PTS content in the K326 cultivar was lowest at the SP site but did not differ among the other three sites. Moreover, the PRS content in K326 did not differ among the four planting sites, whereas the PRS contents in Hongda and Yun87 were higher at ML and SP and lower at JS and LX. Sugar contents were negatively correlated with soil pH, and PRS content was positively correlated with field sunshine hours but negatively correlated with field rainfall. However, both PTS and PRS contents were positively correlated with irrigation water in Guizhou^23^.

#### Effects of environmental factors on sugar/alkaloid and nitrogen/alkaloid ratios in tobacco leaves

Flue-cured tobacco with a high smoke quality has an optimal sugar-to-alkaloid ratio of 6–10, with 10 being the best^55^. A high sugar/alkaloid ratio is associated with mildness, whereas a low sugar/alkaloid ratio is associated with harshness^32^. In our study, the PSA ratio for all tobacco cultivars was > 11, which is markedly above the upper limit of the range for this ratio, and values were lower at the JS and SP sites compared to the ML and LX sites. Furthermore, a PNA ratio closer to 1 is better for the quality of flue-cured tobacco^32^. The PNA ratios were all < 1 in our study, although the average value for the K326 cultivar was closer to 1. The probable cause is that the soil total nitrogen was not suited for forming optimal sugar/alkaloid and nitrogen/alkaloid ratios, similar to high total nitrogen content conditions^29^. These two ratios were positively correlated with field relative humidity and negatively correlated with field temperature in this study. Tobacco is meant to be flavorsome, and its quality is mainly evaluated by smoking^24^. The internal chemical composition of tobacco leaves will also affect the sensory evaluation of tobacco leaves and is influenced by the sugar/alkaloid equilibrium. Specifically, when the internal chemical composition is appropriate, the sensory evaluation score is relatively high.

#### Effects of environmental factors on petroleum ether extract and starch contents in tobacco leaves

Petroleum ether extract and starch determine the quality and appearance rating of tobacco leaves^56,57^. The petroleum ether extract content is influenced by the interactive effects between cultivars and ecological factors^5^. In our study, the petroleum ether extract content was affected by the interactive effects between soil and climatic factors and positively correlated with field sunshine hours and temperature but negatively correlated with field rainfall, field relative humidity, soil clay content, soil pH, and SOM content. On the other hand, when the starch content in tobacco leaves during harvesting is too high, it can cause an imbalance in the chemistry profile by decreasing the nicotine content^58^, thus reducing industrial usability. Overall, the starch content in the Hongda cultivar was the highest and was highest at the LX site. In this study, starch content decreased significantly with increasing temperature and was positively correlated with field rainfall but not influenced by soil properties.

### Effects of climatic and soil factors on the yield and quality of tobacco leaves

The ecological factors that influence the yield and chemical composition of tobacco leaves were divided into two categories: climatic factors and soil physical and chemical properties. Flue-cured tobacco is a chlorine-free crop, and the quality of tobacco leaves is closely related to the carbon and nitrogen metabolism of tobacco plants^50^. The PTS, PRS, PTN, and PN contents can reflect the carbon supply capacity and nitrogen supply capacity^43,52^, whereas the PTK content affects quality^48^, and the PPE and PS contents determine the quality and appearance rating of tobacco leaves^56,57^. Among these indices, the PRS, PN, PTK, and PPE contents were positively correlated with temperature factors but negatively correlated with moisture factors, whereas the PCL and PS contents were negatively correlated with field temperature but positively correlated with field rainfall. On the other hand, although the seven soil physical and chemical properties influenced the five indices related to the internal chemical composition of tobacco leaves (PTS, PRS, PTK, PCL, and PPE), soil pH was a dominant factor that influenced four indices.

RDA results showed that field rainfall, field temperature, and field sunshine hours significantly influenced the yield and quality of the K326, Hongda, and Yun87 cultivars. Similarly, different weather conditions influence the smoking quality of flue-cured tobacco^32^, and tobacco quality is influenced by rainfall^59^. Low rainfall and relative humidity values and high sunshine hours and temperature-related indices are good for producing flue-cured tobacco of high smoking quality in southwestern China^60^. In this study, except for the chlorine and starch contents, the chemical composition in flue-cured tobacco was positively correlated with field temperature and field sunshine hours but negatively correlated with field rainfall and field relative humidity. Further analysis showed that at the LX site, yield and quality in the three cultivars were positively correlated with field rainfall and field relative humidity, but there are obvious differences in drought responses among the different tobacco cultivars^24^. To distinguish the responses of the three cultivars to drought stress, the relevant mechanisms of their stress responses should be further analyzed. The AN content also positively influenced the yield and quality of the K326 cultivar, which is line with reports that the highest tobacco yield can be obtained with high rates of nitrogen application and watering^61^. However, the yield and quality of the Hongda and Yun 87 cultivars were primarily affected by field temperature and field sunshine hours.

The undulating mountains and complex and diverse landforms in the Hani-Yi Autonomous Prefecture of Honghe affect the climactic conditions in this region. Differences in latitude and altitude result in climate variations, and natural conditions differ greatly among sub-regions. The climate is very different among different tobacco-growing sites. The sampling sites belong to different eco-climatic regions to better represent the climate characteristics of the Hani-Yi Autonomous Prefecture of Honghe. Moreover, quality characteristics of tobacco leaves vary widely among different ecological regions. Among the four sampling sites, ML and LX are high-latitude and high-altitude tobacco-growing sites, with the most suitable temperature, a medium level of sunshine hours, and moderate rainfall during the field period, where plants produced higher PTS, PPE and PS contents. JS and SP are low-latitude and low-altitude tobacco-growing sites, with lower rainfall, higher temperature, and longer sunshine hours, where plants produced higher PTN and PN contents. Understanding the variations in characteristics of tobacco leaves among different ecological regions, specifically, as they pertain to which factors influence the quality characteristics of tobacco leaves at each tobacco-growing site, is important for maintaining the stability of and continuously improving local tobacco quality^47,49,52^.

Finally, agronomic practices affect the yield and quality of flue-cured tobacco^5,9,23,44,51^. No special agronomic practices have been adopted at the flue-cured tobacco-planting sites sampled in this study. The amount of fertilizers applied often differs because the basic fertility of the soil and the demand of crops are usually considered when applying fertilizer in the field^21,25,27^. In addition, although the irrigation method used can affect the quality of flue-cured tobacco, the methods are not determined by the cultivars but by the sources of available water^23^. However, the growing time, number of left leaves, picking time, and other characteristics are also strongly correlated with cultivar type. In addition to a discussion on the effects of agronomic practices in different tobacco planting sites on the yield and quality of flue-cured tobacco cultivars in this study, we plan to carry out research in this area in the future to find the most suitable agronomic methods for particular flue-cured tobacco cultivars and provide more scientific guidance for the cultivation and production of local tobacco cultivars. Furthermore, we will also focus on the plant-soil interactions of different cultivars in the same field, which is an important mechanism in the growth of flue-cured tobacco.

## Conclusions

In conclusion, we found 13 variables affecting the yield and internal chemical composition of flue-cured tobacco in this study. Among them, soil properties and climatic factors influenced 7 and 11 variables, respectively. Specifically, soil pH and clay, AN, AP, and SCL contents were the main factors affecting the yield and quality of flue-cured tobacco. In addition, the economic value of flue-cured tobacco was not positively correlated with the number of left leaves or the proportion of superior tobacco. Higher temperatures and longer sunshine hours were beneficial for improving the yield of flue-cured tobacco and can increase the nitrogen and nicotine contents. However, lower temperatures, shorter sunshine hours, and higher rainfall can improve total sugar, petroleum ether, and starch contents. More importantly, the yield and quality of the K326 and Yun87 cultivars were primarily influenced by moisture factors, whereas the yield and quality of the Hongda cultivar was primarily affected by temperature factors. These results demonstrate that climatic factors were the main factors affecting the yield and quality of flue-cured tobacco.

## Data Availability

All data generated or analyzed during this study are included in this article.
